# Highly efficient bioconversion of icariin to icaritin by whole-cell catalysis

**DOI:** 10.1186/s12934-023-02068-4

**Published:** 2023-04-04

**Authors:** Yu Lin, Wen-wen Chen, Bo Ding, Man Guo, Meng Liang, Hao Pang, Yu-tuo Wei, Ri-bo Huang, Li-qin Du

**Affiliations:** 1 State Key Laboratory for Conservation and Utilization of Subtropical Agro-bioresources, Guangxi Research Center for Microbial and Enzymatic Technology, College of Life Science and Technology, Guangxi University, Daxue Road No. 100, Nanning, 530005 Guangxi China; 2 Guangxi Key Laboratory of Bio-refinery, National Engineering Research Center for Non-Food Biorefinery, State Key Laboratory of Non-Food Biomass and Enzyme Technology, Guangxi Academy of Sciences, Daling Road No. 98, Nanning, 530007 Guangxi China

**Keywords:** Icariin, Icaritin, Whole-cell catalysis, α-L-rhamnosidase, β-glucosidase

## Abstract

**Background:**

Icaritin is an aglycone of flavonoid glycosides from *Herba Epimedii*. It has good performance in the treatment of hepatocellular carcinoma in clinical trials. However, the natural icaritin content of *Herba Epimedii* is very low. At present, the icaritin is mainly prepared from flavonoid glycosides by α-L-rhamnosidases and β-glucosidases in two-step catalysis process. However, one-pot icaritin production required reported enzymes to be immobilized or bifunctional enzymes to hydrolyze substrate with long reaction time, which caused complicated operations and high costs. To improve the production efficiency and reduce costs, we explored α-L-rhamnosidase SPRHA2 and β-glucosidase PBGL to directly hydrolyze icariin to icaritin in one-pot, and developed the whole-cell catalytic method for efficient icaritin production.

**Results:**

The SPRHA2 and PBGL were expressed in *Escherichia coli*, respectively. One-pot production of icaritin was achieved by co-catalysis of SPRHA2 and PBGL. Moreover, whole-cell catalysis was developed for icariin hydrolysis. The mixture of SPRHA2 cells and PBGL cells transformed 200 g/L icariin into 103.69 g/L icaritin (yield 95.23%) in 4 h in whole-cell catalysis under the optimized reaction conditions. In order to further increase the production efficiency and simplify operations, we also constructed recombinant *E. coli* strains that co-expressed SPRHA2 and PBGL. Crude icariin extracts were also efficiently hydrolyzed by the whole-cell catalytic system.

**Conclusions:**

Compared to previous reports on icaritin production, in this study, whole-cell catalysis showed higher production efficiency of icaritin. This study provides promising approach for industrial production of icaritin in the future.

**Supplementary Information:**

The online version contains supplementary material available at 10.1186/s12934-023-02068-4.

## Background

**Fig. 1:**
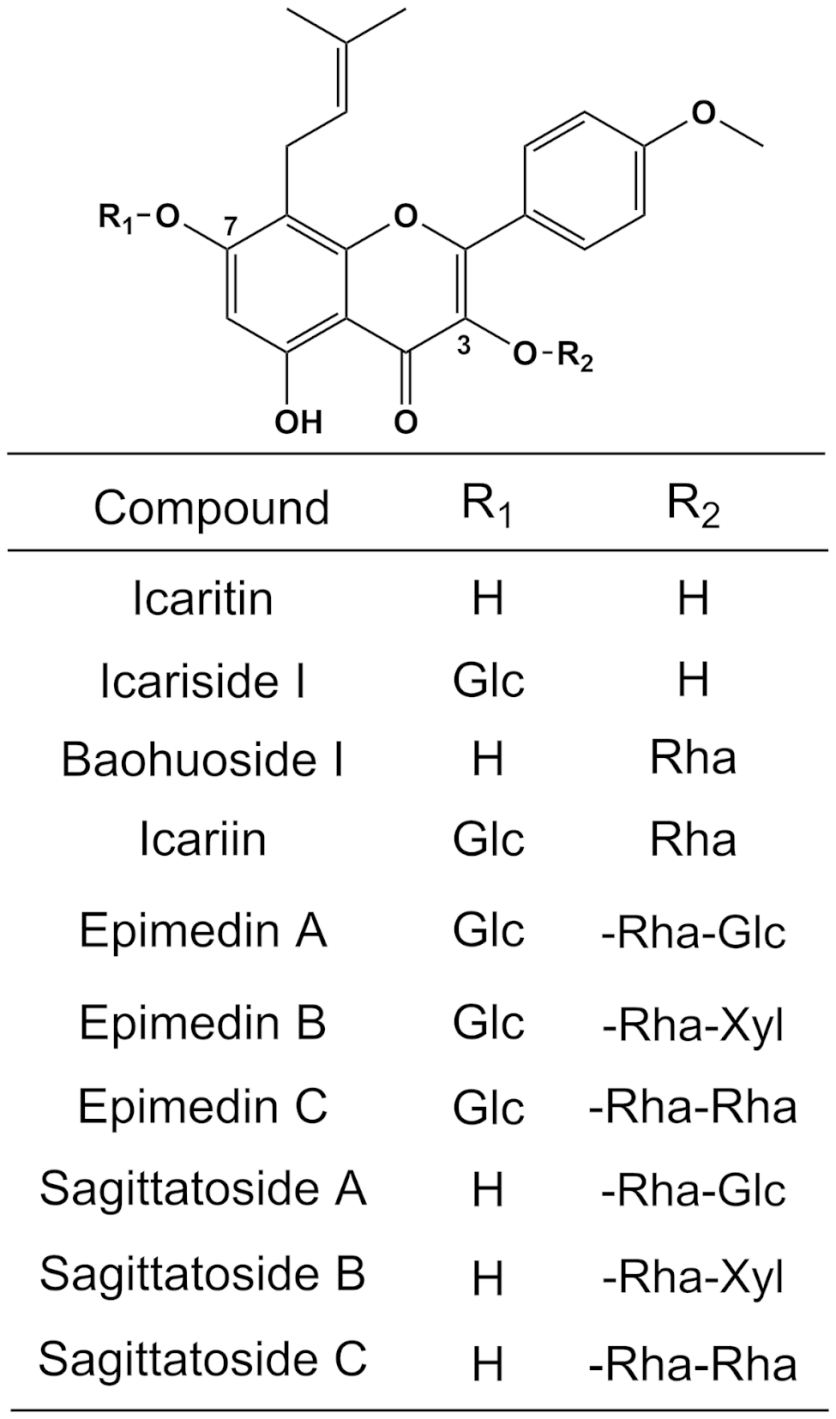
Structures of flavonoid glycosides. Glc: glucoside; Rha: Rhamnoside; Xyl: xyloside

*Herba Epimedii* (Yinyanghuo in Chinese), the traditional Chinese medicine, has been used in China for more than 2,000 years [1]. It has proven anti-osteoporotics [2], anti-cancer [3], anti-inflammatory activities [4] and reproductive functions [5]. The main active substances in *Herba Epimedii* are flavonoid glycosides, including icariside I, baohuoside I, epimedin A, B, C and icariin [6]. They are formed by varying degrees of glycosylation of the C-3 and C-7 positions of icaritin (Fig. 1) [7].

Icaritin is an aglycone of flavonoids without any glycosylation. It has higher bioactivity than the other flavonoid glycosides in the treatment of some diseases [8, 9]. In addition, it enhanced stem cell proliferation, migration and osteogenic differentiation [10] and had a powerful effect in treatment of multiple cancers [11]. Clinical trials of icaritin indicated that it has good performance in treatment of hepatocellular carcinoma [12, 13]. However, the natural icaritin content of *Herba Epimedii* is very low. The icariin is one of considerable components in extracts of *Herba Epimedii* [14, 15]. Icariin has rhamnosidic linkage and β-glucosidic linkage at the C-3 and C-7 positions, respectively. Thus, α-L-rhamnosidases and β-glucosidases have been widely applied in icaritin production. However, the reaction conditions of α-L-rhamnosidases and β-glucosidases are incompatibility, and thus a two-step process is usually used [16–18], which restricts the production efficiency of icaritin. In addition, the icariin is difficult to dissolve in water, which affected the efficiency of enzymatic catalysts. Therefore, exploring one-step enzymatic catalysts and hydrolyzing high concentration of substrate have the potential to improve icaritin production.

Immobilization of α-L-rhamnosidases and β-glucosidases has been reported in hydrolysis of Epimedii flavonoids. Immobilization of the thermostable enzyme 1000NH-DthRha and 1000NH-DthBgl3 was reported for hydrolysis of Epimedii flavonoids to icaritin [19], the molar conversion rate was 87.21%, but the substrate concentration was only 10 g/L. Recently, co-immobilization of α-L-rhamnosidase (Rha1) and β-glucosidase (Glu4) using cross-linked enzyme aggregates was reported to achieve one-pot production of icaritin by hydrolyzing a high concentration of epimedin C. However, the icaritin yield was only 77.45% at an epimedin C concentration of 100 g/L [20]. Although immobilized enzymes have higher stability and they can be reused, the operation processes are very complicated, which is a significant drawback for industrial application. Thus, further exploring efficient method is still important for icaritin production. Whole-cell catalysis omits isolation and purification of enzymes and reduces costs. The cells can provide a natural, protective environment for the enzymes [21]. However, few reports focus on hydrolysis of flavonoid glycosides by whole-cell catalysis. A strain of *Aspergillus niger* with α-L-rhamnosidase and β-glucosidase activities has been reported, it efficiently hydrolyzed flavonoid glycosides by whole-cell catalysis, including hesperidin, rutin, naringin, neohesperidin and naringin dihydrochalcone [22]. Whole-cell catalysis using resting cells to completely hydrolyze epimedin C to icariin following expression of α-L-rhamnosidase in *E. coli* has also been reported [23]. However, to our knowledge, whole-cell catalysis icariin into icaritin has not yet been reported.

In this work, α-L-rhamnosidase SPRHA2 from *Novosphingobium* sp. GX9 and β-glucosidase PBGL from *Paenibacillus cookii* GX-4 were cloned and identified to hydrolyze icariin to produce icaritin in one-pot enzymatic method. Moreover, whole-cell catalysis for icariin hydrolysis was investigated. The results showed whole-cell catalytic system had better performance in hydrolysis of high purity icariin and crude icariin extracts. This study have potential for use in industrial production of icaritin.

## Results

### Purification and basic analysis of SPRHA2 and PBGL

Icariin has rhamnosidic linkage and β-glucosidic linkage at the C-3 and C-7 positions, respectively. Icaritin can thus be produced by hydrolysis of icariin by α-L-rhamnosidases and β-glucosidases. The hydrolysis of icariin was investigated by α-L-rhamnosidase SPRHA2 and β-glucosidase PBGL. The ORF of *sprha2* was 3,498 bp. Analysis results from SMART [24] revealed that SPRHA2 contains a glycoside hydrolase (GH) domain that belongs to GH family 106 and a GH 2 N domain (sugar binding domain). Prediction using SignalP-5.0 [25] indicated that amino acids 1 to 31 of SPRHA2 form a signal peptide. The sequence coding signal peptide was removed by PCR for recombinant intracellular expression in *E. coli*. The ORF of *pbgl* was 2,280 bp. PBGL contains two GH family 3 domains (residues 107 to 465, and 504 to 744) and did not have a predicted signal peptide.

**Fig. 2:**
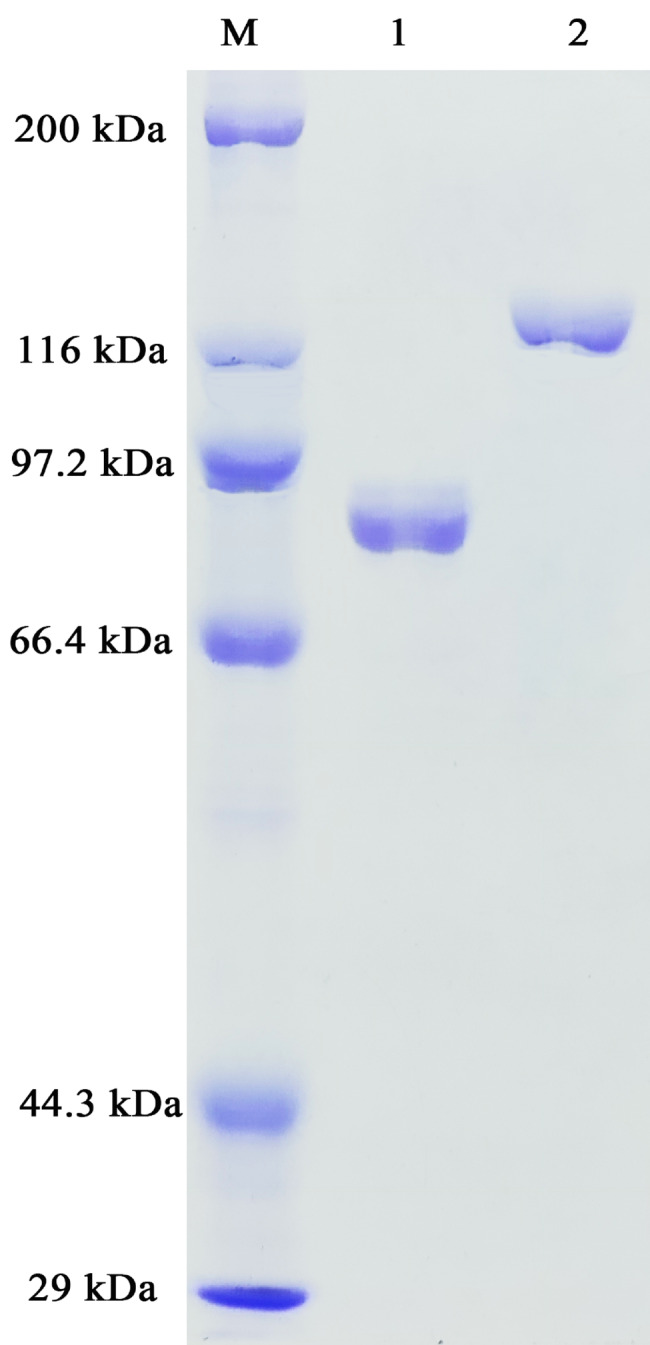
10% SDS-PAGE analysis of purified SPRHA2 and PBGL. M: protein marker; lane 1: purified PBGL (~ 84 kDa); lane 2: purified SPRHA2 (~ 120 kDa)

The purified recombinant SPRHA2 and PBGL were analyzed by SDS-PAGE. The molecular weights of SPRHA2 and PBGL were ~ 120 kDa and ~ 84 kDa (Fig. 2), respectively. *K*_m_ and *V*_max_ values of SPRHA2 and PBGL were determined using the substrates *p*NPR and *p*NPG, respectively: SPRHA2, *K*_m_: 0.63 ± 0.02 mM, *V*_max_: 267.60 ± 2.86 µmol min^-1^ mg^-1^; PBGL, *K*_m_: 0.17 ± 0.02 mM, *V*_max_: 149.00 ± 3.40 µmol min^-1^ mg^-1^.

### Characterization of the enzymes in hydrolysis of flavonoid glycosides

**Fig. 3:**
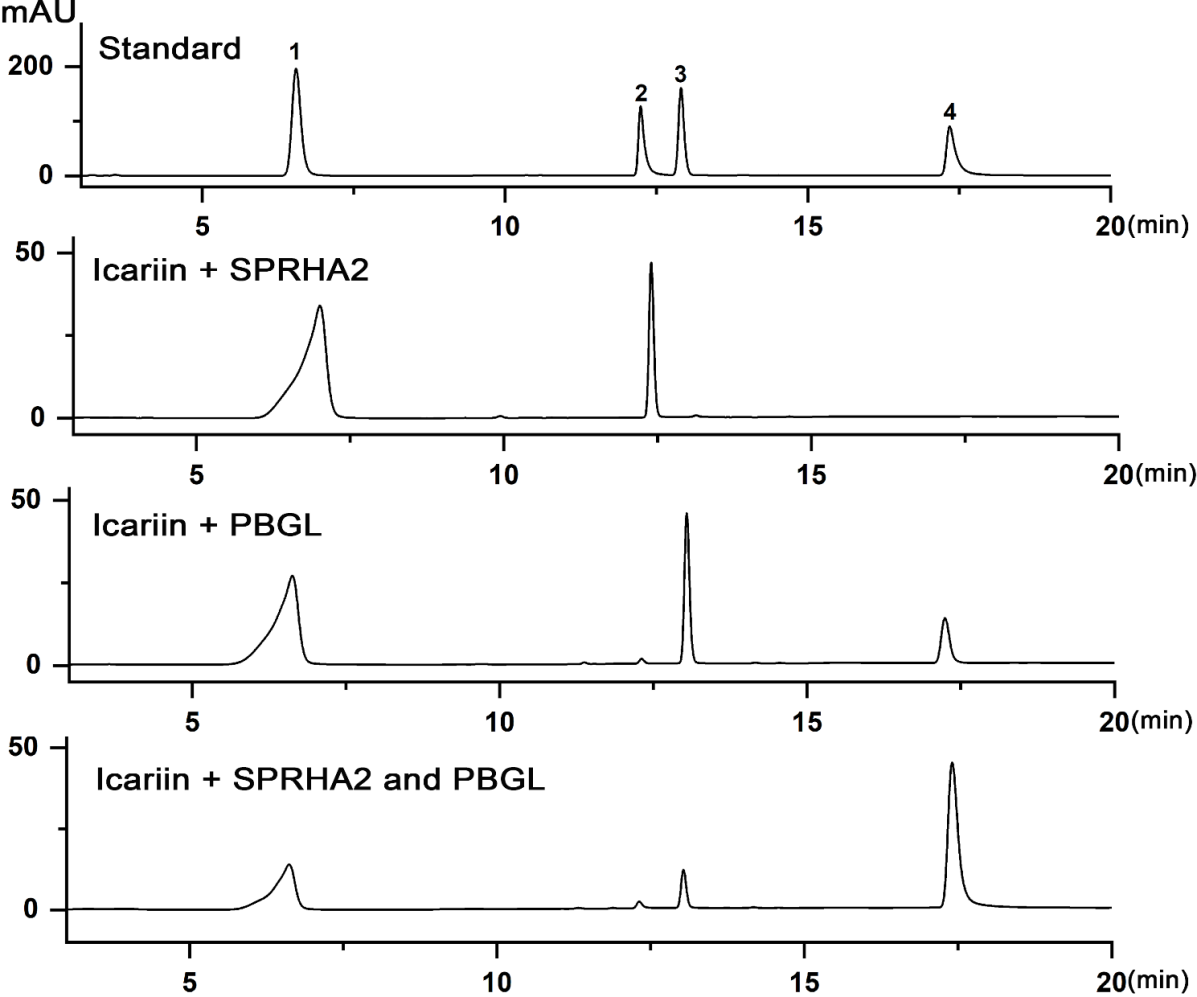
HPLC analysis of icariin hydrolysis by SPRHA2 and PBGL. Peak 1: icariin; peak 2: icariside I; peak 3: baohuoside I; peak 4: icaritin

**Fig. 4:**
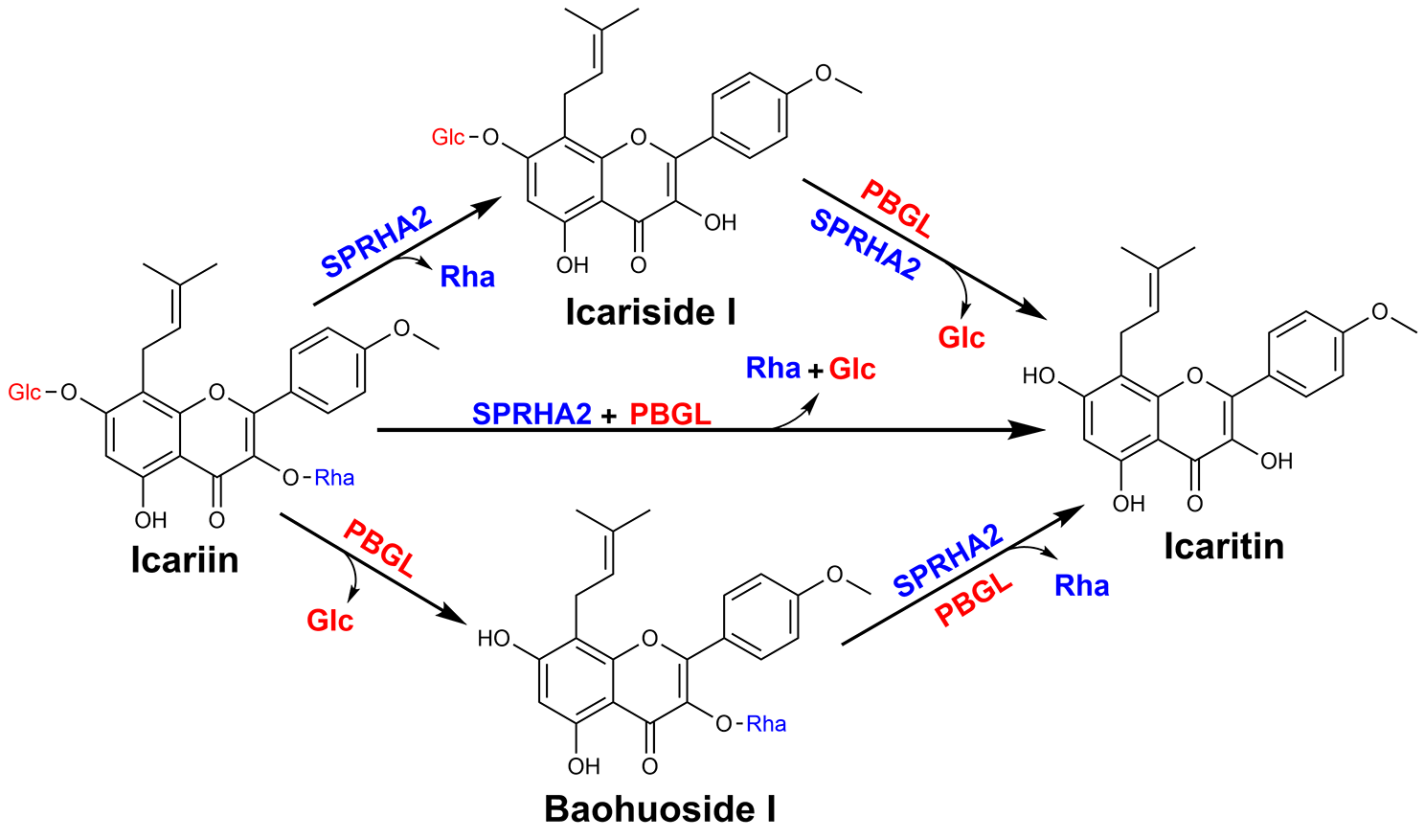
Scheme of biotransformation pathways for icaritin preparation from icariin by SPRHA2 and PBGL

In this study, co-hydrolyzed icariin by α-L-rhamnosidase SPRHA2 and β-glucosidase PBGL to produce icaritin was assayed. First, the enzymes were applied individually to catalyze icariin. HPLC analysis results showed that the α-L-rhamnosidase SPRHA2 catalyzed icariin into icariside I (Fig. 3). Surprisingly, β-glucosidase PBGL converted icariin into baohuoside I and icaritin, exhibiting α-L-rhamnosidase activity, but icariside I was not found. Next, hydrolysis of the intermediates icariside I and baohuoside I was studied to further determine the properties of SPRHA2 and PBGL. The results suggested that SPRHA2 catalyzed conversion of both icariside I and baohuoside I to icaritin, but it showed low activity toward icariside I. PBGL could catalyze conversion of icariside I and baohuoside I to icaritin (Additional file 1: Figure S1). Thus, these data indicate that SPRHA2 and PBGL are bifunctional enzyme. Either SPRHA2 or PBGL has the ability to hydrolyze icariin to icaritin. In a previous report, an Epimedii flavonoid-glycosidase from *Aspergillus* sp. y848 had the same effect in hydrolysis of icariin as PBGL [26]. The effect of combination of SPRHA2 and PBGL on the hydrolysis of icariin to produce icaritin was better than that of SPRHA2 or PBGL alone. Detailed transformation pathways are shown in Fig. 4.

The hydrolysis of epimedin A, B and C by SPRHA2 and PBGL was also studied. The results showed that epimedin A was converted into sagittatoside A, a small amount of baohuoside I and icaritin by PBGL. PBGL could cleave the β-glucosidic linkages of epimedin B and C, to respectively form sagittatoside B and C, but could not break the rhamnosidic linkages at the C-3 position (Additional file 1: Figure S2). Epimedin A and B were respectively converted into sagittatoside A and B by SPRHA2, but the activity was lower than that of PBGL. These data confirmed that SPRHA2 could hydrolyze the β-glucosidic bond at position C-7. In addition, SPRHA2 catalyzed epimedin C into sagittatoside C, icarisde I and icaritin, but icariin was not detected. To determine if icariin was produced in the reaction, the time course of epimedin C hydrolysis by SPRHA2 was studied. The results confirmed that icariin was not a product (Additional file 1: Figure S3). We speculate that SPRHA2 simultaneously hydrolyzed the β-glucosidic linkage at C-7 and two α-L-rhamnosidic linkages at C-3 to produce sagittatoside C and icariside I, respectively.

### Assay of icariin co-hydrolysis by SPRHA2 and PBGL

**Fig. 5:**
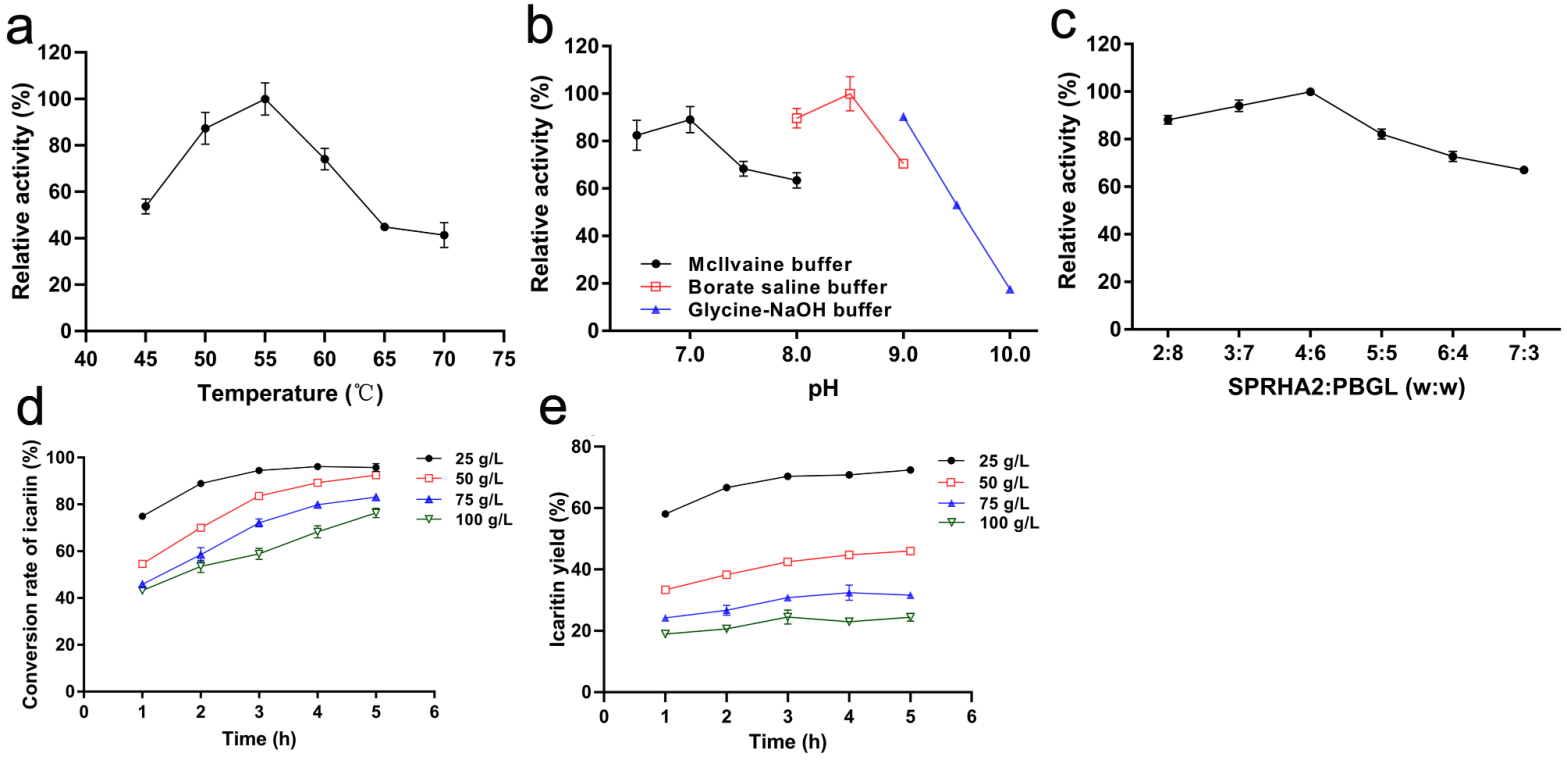
Co-hydrolysis of icariin by SPRHA2 and PBGL. (a) Effect of temperature. (b) Effect of pH. (c) Effect of the ratio of SPRHA2 and PBGL (w:w). (d) Effect of time on the conversion rate of icariin. (e) Effect of time on the icaritin yield

The influence of temperature and pH on co-hydrolysis of icariin by SPRHA2 combined with PBGL was investigated. The optimal temperature for the reaction was 55 °C (Fig. 5a), and the highest activity was observed in 200 mM borate saline buffer at pH 8.5 (Fig. 5b). This is the first report of hydrolysis of icariin in alkaline environment. It was worth mentioning that compared with one-pot catalytic method, the temperature and pH of the reaction after the first step should be adjusted to satisfy the needs of the second step in two-step catalytic methods; this increases energy consumption and processing costs. Here, we developed a one-pot process that did not require adjustment of the conditions during the reaction, which is advantageous for icaritin production.

To increase the production efficiency of icaritin and lower the amount of residual intermediates, the optimum weight ratio of SPRHA2 and PBGL was assessed. Reactions were performed with 0.2 g/L icariin and different weight ratios of the enzymes (total protein concentration: 10 mg/L) at 55 °C, pH 8.5 for 10 min. The results showed that optimum weight ratio of SPRHA2 to PBGL was 4:6 (w:w) by HPLC analysis (Fig. 5c). Finally, in the optimum reaction conditions determined in this study, high concentrations of icariin were co-hydrolyzed by SPRHA2 and PBGL. Icariin is difficult to dissolve in water, and solubility of its hydrolysis products is even lower, which hinders the effective hydrolysis of icariin. Therefore, the reactions were shaken to improve the efficiency of icariin hydrolysis. The different concentrations of icariin were catalyzed by 40 mg/L SPRHA2 and 60 mg/L PBGL at 55 °C and pH 8.5 with shaking at 220 rpm for 5 h. The conversion rate of icariin decreased with the increase of substrate concentration (Fig. 5d). After 5 h, the icaritin yield was 72.43% when the substrate icariin concentration was 25 g/L; at higher substrate concentrations, the yields were below 50% (Fig. 5e). Thus, the co-hydrolysis system of icariin by purified SPRHA2 and PBGL does not have good performance for icaritin production.

### Whole-cell catalysis for icaritin production by co-hydrolysis

**Fig. 6:**
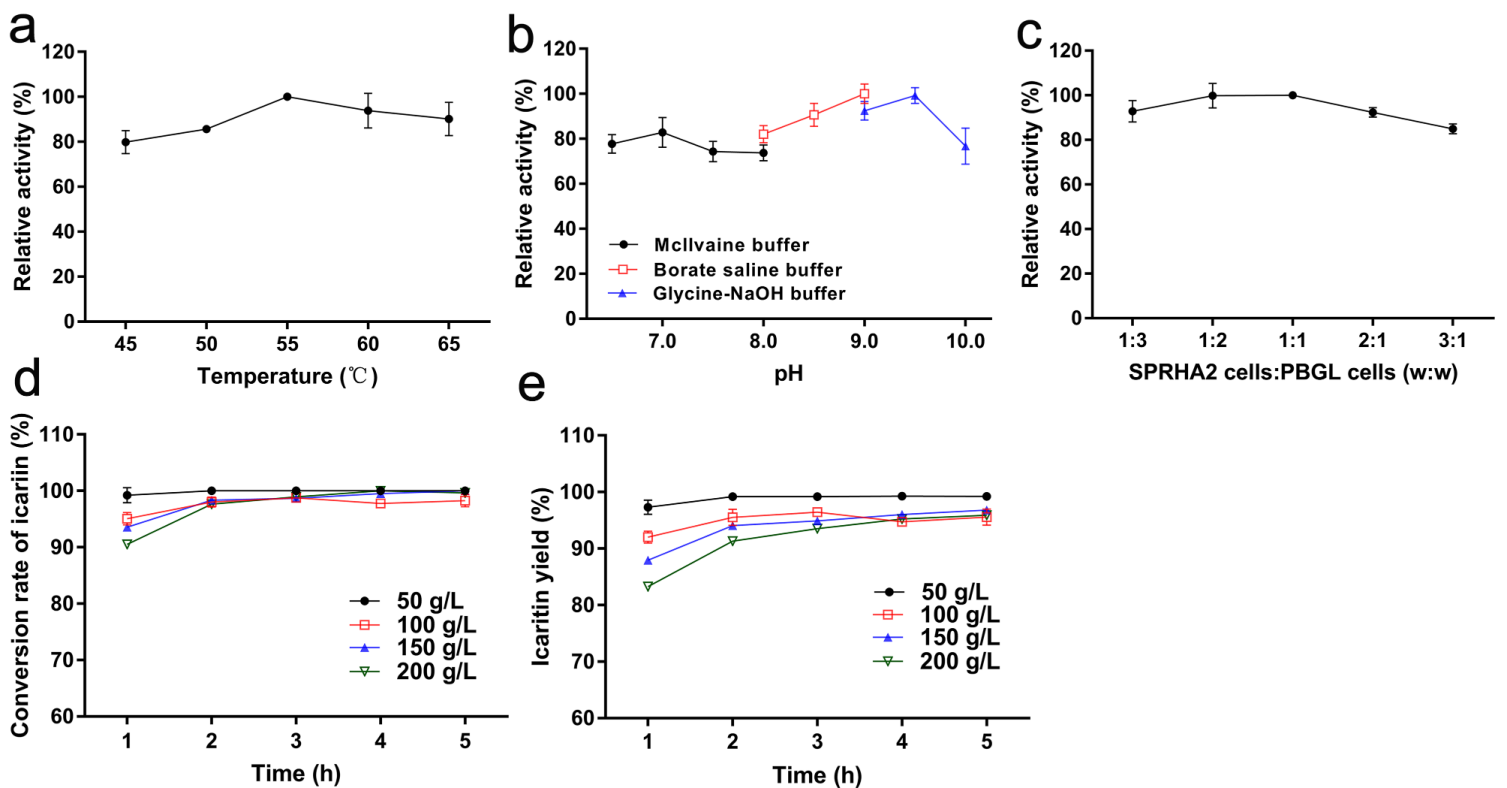
Whole-cell co-hydrolysis of icariin by SPRHA2 cells and PBGL cells. (a) Effect of temperature. (b) Effect of pH. (c) Effect of the weight ratio of SPRHA2 cells and PBGL cells. (d) Effect of time on the conversion rate of icariin. (e) Effect of time on the icaritin yield

To increase the production efficiency of icaritin and reduce costs, the whole-cell catalysis was examined in this study. The reaction properties were studied on co-hydrolysis of icariin by mixtures of *E. coli* cells harboring plasmids pET-*sprha2* and pET-*pbgl* respectively. The optimal temperature and pH of whole-cell catalysis were determined to be 55 °C and 9.0 (Fig. 6a and b), respectively. The optimum pH for the whole-cell catalysis was thus more alkaline than that for the purified enzymes, and it exhibited high activity even at pH 9.5. The optimal temperature was not changed compared with that for the purified enzymes.

The optimal weight ratio of wet cells containing SPRHA2 and PBGL, respectively, was investigated. The reaction system (200 µL) contained 1 g/L icariin and different ratios of wet cells (Total wet cell weight: 5 g/L); it was incubated at 55 °C, pH 9.0 with shaking at 220 rpm for 10 min. The results showed that optimal weight ratio was 1:1 (Fig. 6c).

The time course of whole-cell hydrolysis of icariin under optimal conditions was studied to analyze catalytic process. Icariin was completely converted into icaritin in 2 h. Baohuoside I was the only intermediate. Icariside I was not detected (Additional file 1: Figure S4).

Due to icariin is difficult to dissolve in water, the enzymatic reaction at high concentration of substrate may be insufficient. Thus we tested multiple concentrations of substrate icariin in whole-cell catalysis. When reactions contained 20 g/L wet weight SPRHA2 cells and 20 g/L wet weight PBGL cells (incubated at 55 °C, pH 9.0 with shaking at 220 rpm for 5 h), 50 g/L icariin was completely hydrolyzed within 1 h, and the conversion rates of the icariin (100–200 g/L) were more than 90% by this time (Fig. 6d). The 200 g/L icariin was completely hydrolyzed after 4 h, producing 103.69 g/L icaritin with a yield of 95.23% (Fig. 6e). The product icaritin was confirmed by NMR analysis (Additional file 1: Table S1).

**Fig. 7:**
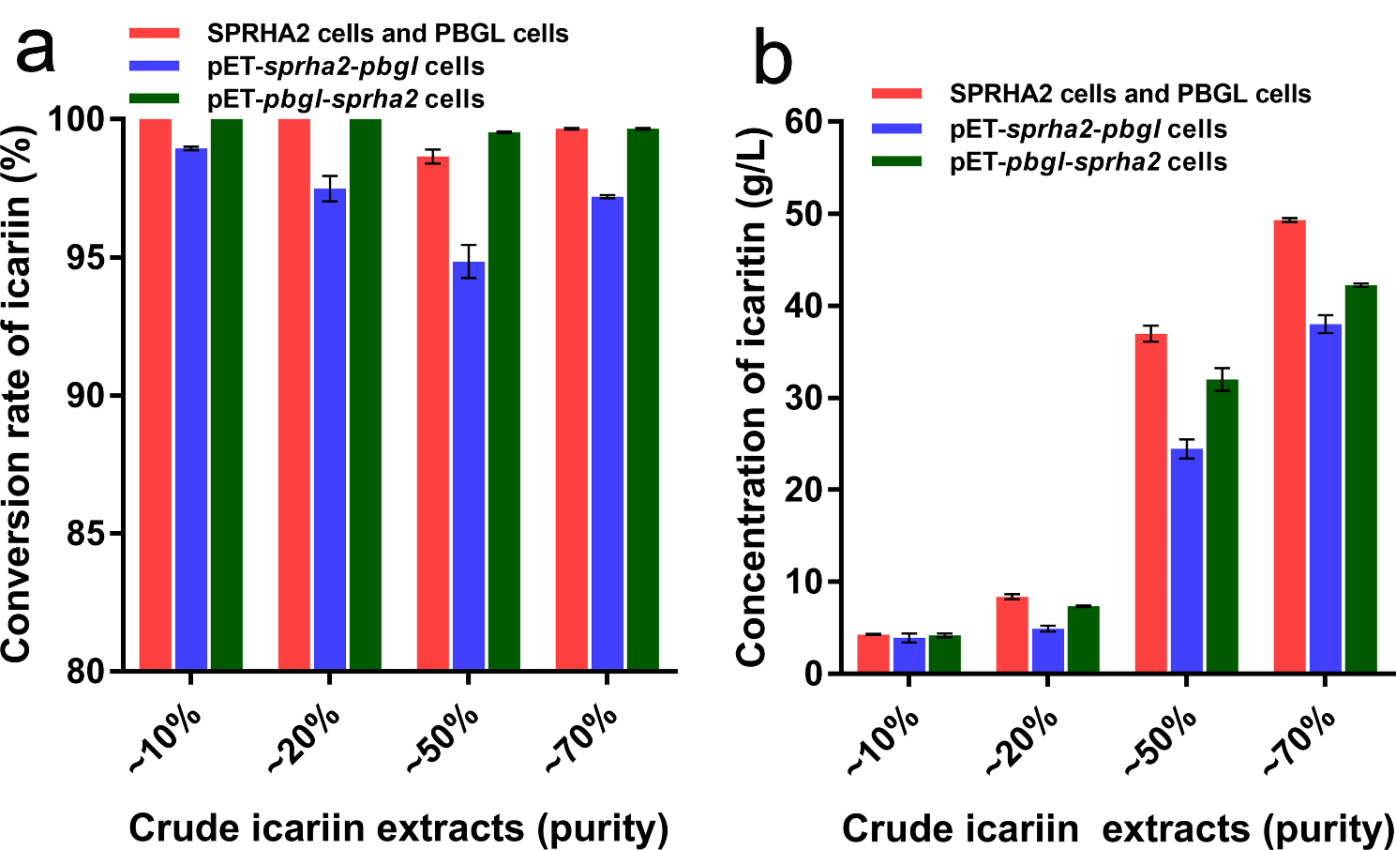
Effect of expression patterns of SPRHA2 and PBGL on whole-cell catalysis of crude icariin extracts. (a) Conversion rate of icariin in the different purity of substrate. (b) Concentration of icaritin produced in the different purity of substrate

High-purity substrates are more expensive as raw materials, therefore crude icariin extracts were selected (purity ~ 10%, ~ 20%, ~ 50%, and ~ 70%) and we assessed their hydrolysis in whole-cell catalysis. The reaction system (500 µL) contained 200 g/L icariin crude extract, 20 g/L wet weight SPRHA2 cells and 20 g/L wet weight PBGL cells, and was incubated at 55 °C, pH 9.0 with shaking at 220 rpm for 4 h. The results indicated that all conversion rates of icariin were more than 98% (Fig. 7a), 4.27, 8.37, 36.96 and 49.31 g/L icaritin was respectively obtained by hydrolysis of crude icariin extracts (Fig. 7b). However, compared with hydrolysis of 98% pure icariin, the conversion rate of icariin was slightly lower. We speculate that crude icariin extracts contain some unknown impurities that hampered the reactions in whole-cell catalysis.

### Whole-cell catalyzed conversion of crude icariin extracts into icaritin by co-expression

Whole-cell catalysts can execute multiple reactions by assembling enzymes [27]. Next, we attempted to co-express SPRHA2 and PBGL in *E. coli* to further simplify operations. The plasmids pET-*sprha2*-*pbgl* and pET-*pbgl*-*sprha2* were constructed. The genes *sprha2* and *pbgl* shared one T7 promoter and had separate ribosome-binding sites. The reaction system (500 µL) contained 200 g/L icariin crude extract and 40 g/L wet weight cells of the co-expression strain; the reaction mixture was incubated at 55 °C, pH 9.0 with shaking at 220 rpm for 4 h. The results showed that conversion rate of icariin by the strain harboring plasmid pET-*pbgl*-*sprha2* was more than 99%, which was higher than for the strain harboring plasmid pET-*sprha2*-*pbgl* (Fig. 7a), suggesting that co-expression plasmid pET-*pbgl*-*sprha2* was more suitable for icariin hydrolysis than plasmid pET-*sprha2*-*pbgl*. The protein expression levels of the two plasmid constructions were analyzed by SDS-PAGE. The results showed that the PBGL expression level in the pET-*pbgl*-*sprha2* was significant higher than that in the pET-*sprha2*-*pbgl*. The total protein content of these two enzymes was also higher under the plasmid pET-*pbgl*-*sprha2* condition (Additional file 1: Fig. S5). The product icaritin concentrations from strain harboring plasmid pET-*pbgl*-*sprha2* were 4.16, 7.35, 31.99 and 42.22 g/L from crude icariin extracts, respectively (Fig. 7b), lower than when SPRHA2 and PBGL were expressed in separate strains. HPLC analysis showed that more residual intermediates were produced in the strains co-expressing both SPRHA2 and PBGL than expression of individual genes. The strain with plasmid pET-*pbgl*-*sprha2* had good performance in hydrolysis of crude icariin extracts, its icaritin yield was more than 85% of the yield of mixture cells on hydrolysis of ~ 50% and ~ 70% pure crude icariin extracts. Expression of multiple genes may cause a greater burdens on *E. coli* than expression of individual genes [28], which may explain why the strains co-expressing both PBGL and SPRHA2 were not as effective as was mixing cells of the strains individually expressing these enzymes.

## Discussion

To date, many studies have been performed the flavonoid glycosides hydrolysis by enzymatic catalysis for icaritin production. The flavonoid glycosides mainly include epimedin C and icariin. Therefore, α-L-rhamnosidases and β-glucosidases were widely applied in icaritin production. However, the reaction conditions of α-L-rhamnosidases and β-glucosidases are incompatibility, thus a two-step catalysis process is usually used [17, 18, 29]. In previous report, the α-L-Rhamnosidase Rhase-I from *Talaromyces stollii* CLY-6 was expressed in *P. pastoris*, then Rhase-I and β-glucosidase Bglsk from *Sanguibacter keddieii* DSM 10,542 hydrolyzed epimedin C in two-step, 20 g/L of epimedin C was converted into 8.83 g/L of icaritin with a yield of 98.66% [16]. To simplify operations and reduce costs, it is important to developed one-step catalysis. At present, the one-step catalysis was achieved by the bifunctional enzymes or immobilized enzymes. A glycosidase from *Aspergillus* sp.y848 has α-L-rhamnosidase and β-glucosidase activities, and it directly hydrolyzed icariin to icaritin, the 5.04 g icaritin with 98% purity was obtained from 10 g icariin in 18–20 h [26]. However, the reaction time was so long. The α-L-rhamnosidase Rha1 and β-glucosidase Glu4 were co-immobilized by cross-linked enzyme aggregates, the 34.24 g/L icaritin was obtained from 100 g/L epimedin C in one-pot catalysis within 8 h [20]. But the immobilization of enzymes has complicated operations. In this study, one-step catalysis of icariin was achieved by SPRHA2 and PBGL without any modification. In addition, PBGL could directly hydrolyze icariin to produce icaritin. But SPRHA2 and PBGL co-hydrolyzed icariin with better performance than PBGL alone. Next, the reaction conditions of icariin co-hydrolysis were optimized. The 9.85 g/L icaritin was produced from 25 g/L icariin in one-step in 5 h, and the yield was 72.43%. However, this productivity of icaritin by co-hydrolysis of SPRHA2 and PBGL was not as high as two-step catalysis and immobilized enzymes hydrolysis (Table 1). In addition to icaritin production by hydrolysis, engineered *Saccharomyces cerevisiae* and *E. coli* were built with complete icaritin biosynthesis pathways, 19.7 mg/L icaritin was obtained in co-culture [30]. However, this productivity was lower than reported hydrolysis methods.

**Table 1:** Comparison of icaritin production using different enzymes and reaction conditions

Organism(s)	Enzyme(s)	Substrate	Reaction conditions (type, temperature, pH, time)	Icaritin (g/L)	Yield rate (%)	References
*Novosphingobium* sp. GX9 and *Paenibacillus cookii* GX-4	α-L-rhamnosidase and β-glucosidase	25 g/L icariin	One-pot enzymatic reaction, 55 °C, pH 8.5, 5 h	9.85	72.43%	This study
	α-L-rhamnosidase and β-glucosidase	200 g/L icariin	Whole-cell catalysis, 55 °C, pH 9.0, 4 h	103.69	95.23%	This study
*Talaromyces stollii* CLY-6 and *Sanguibacter keddieii* DSM 10,542	α-L-rhamnosidase and β-glucosidase	20 g/L epimedin C	Two-step enzymatic reaction, 1_st_: 45 °C, pH 4.5, 40 min, 2_nd_: 30 °C, pH 7.5, 20 min	8.85	98.66%	[16]
*Thermotoga petrophila* DSM 13,995	α-L-rhamnosidase and β-glucosidase	1 g/L epimedin C	Two-step enzymatic reaction, 1_st_: 90 °C, pH 4.5, 100 min; 2_nd_: 90 °C, pH 4.5, 50 min	0.43	96.90%	[29]
*Dictyoglomus thermophilum* DSM3960	α-L-rhamnosidase and glucosidase	5 g/L TFE^a^	Two-step enzymatic reaction, 1_st_: 80 °C, pH 5.5, 2 h; 2_nd_: 80 °C, pH 5.5, 2 h	0.21	64.54%	[17]
*Dityoglomus thermophilum* DSM3960	Immobilized α-L-rhamnosidase and β-glucosidase	10 g/L TFE	One-pot enzymatic reaction, 85 °C, pH 6.0, 2 h	0.57	87.21%	[19]
*Aspergillus* sp.y848	Glycosidase	10 g/L icariin	One-pot enzymatic reaction, 40 °C, pH 5.0, 18–20 h	5.04	92.50%	[26]
*Talaromyces stollii* CLY-6	Immobilized α-L-rhamnosidase and β-glucosidase	100 g/L epimedin C	One-pot enzymatic reaction, 55 °C, pH 5.0, 8 h	34.24	77.45%	[20]
*Aspergillus terreus* CCF3059 and *Thermotoga thermarum* DSM5069	α-L-rhamnosidase and β-glucosidase	0.5 g/L icariin	Two-step enzymatic reaction, 1_st_: 65 °C, pH 5.5, 6 h; 2_nd_: 85 °C, pH 5.5, 80 min	0.25	91.20%	[18]

^a^TFE: total flavonoid extract, including epimedin A, epimedin B, epimedin C, icariin and baohuoside I

To improve the production efficiency of icaritin and reduce costs, we explored the whole-cell catalysis for icariin hydrolysis. The combination of SPRHA2 cells and PBGL cells in icariin hydrolysis was investigated. The optimum pH for the whole-cell catalysis was 9.0, and it exhibited high activity even at pH 9.5. However, most reported enzymes hydrolyzed flavonoid glycosides with a pH below 6.0 [17, 18, 29]. It was first report on hydrolysis of flavonoid glycosides in high alkaline. Moreover, a very high concentration of substrate was hydrolyzed. The 200 g/L icariin was completely co-hydrolyzed by SPRHA2 cells and PBGL cells in 4 h, producing 103.69 g/L icaritin with a yield of 95.23%. Obviously, with high concentrations of substrate conditions, whole-cell catalysis exhibited superior performance in icaritin production with the final concentration was 10.5 times higher than purified SPRHA2 and PBGL (Table 1). The reason is that the enzymes may be more stable inside cells [31]. In previous report, the highest concentration of substrate by hydrolysis was 150 g/L epimedin C. However, the yield of icaritin was below 80% [20]. To our knowledge, this is the first report of flavonoid glycosides hydrolysis at such a high concentration (200 g/L) by whole-cell catalysis, and the highest concentration of icaritin produced. In order to further increase the production efficiency and simplify operations, we also constructed recombinant *E. coli* strains that co-expressed SPRHA2 and PBGL for whole-cell catalysis. And crude icariin extracts, as cheap raw materials, were also effectively hydrolyzed by the whole-cell catalytic system. Thus this study has great potential to meet the need of industrial icaritin production.

## Conclusions

In this study, the bioconversion of icariin to icaritin using α-L-rhamnosidase SPRHA2 from *Novosphingobium* sp. GX9 and β-glucosidase PBGL from *Paenibacillus cookii* GX-4 was assayed. One-pot production of icaritin was achieved. Moreover, we developed a whole-cell catalytic method for icaritin production, which exhibited higher production efficiency. 200 g/L icariin was completely hydrolyzed by whole-cell catalysis in 4 h; icaritin yield was 95.23% (103.69 g/L). Recombinant strains were also constructed that co-expressed SPRHA2 and PBGL for whole-cell catalysis. Crude icariin extracts were efficiently transformed to icaritin in whole-cell catalysis. This study provides an efficient method for industrial icaritin production.

## Materials and methods

### Materials, chemicals and strains

Icariin (purity 98.0%) and baohuoside I (purity 98.0%) were purchased from Shanghai Aladdin Bio-chemical Technology Co., Ltd. (Shanghai, China). Icariside I (purity 98.0%), icaritin (purity 99.0%), epimedin A, B and C (purity 98.0%) were purchased from Shanghai Yuanye Bio-Technology Co., Ltd. (Shanghai, China). Crude icariin extracts (purity ~ 10%, ~ 20%, ~ 50% and ~ 70%) were purchased from Shanxi Sunrun Bio-tech. Co., Ltd. (Shanxi, China). All restriction enzymes, PrimeSTAR DNA polymerase, T4 DNA ligase and In-Fusion HD Cloning kit were purchased from Takara Co., Ltd. (Dalian, China). *E. coli* DH5α was used for plasmid propagation and construction. *E. coli* BL21 (DE3) was used for expression of heterologous genes.

### Plasmid construction

The coding sequences of SPRHA2 (GenBank accession: QGA89207.1) and PBGL (GenBank accession: OP810493) were amplified by PCR from genomic DNA of *Novosphingobium* sp. GX9 and *Paenibacillus cookii* GX-4, respectively. The primers were as follows:

sprha2-1: 5′-CAGGGATCCGAGCCGGCACCCGATGCGGCCGC-3′;

sprha2-2: 5′-CAGAAGCTTTCAGCGGGTCGTGCCCAGCGTGAC-3′;

pbgl-1: 5′-CGCGGATCCAGAAACCATACTTCAGACACGATCAA-3′; and pbgl-2: 5′-CGCAAGCTTTCAGCTTCTACGGTATTTCTTGGTTC-3′.

The DNA fragments were digested by restriction enzymes *Bam*HI and *Hin*d III and ligated by T4 ligase into plasmid pET30a digested with the same restriction enzymes, resulting in plasmids pET-*sprha2* and pET-*pbgl*, respectively. The recombinant plasmids were verified by DNA sequencing (Ruibo Tech, Guangzhou, China). The plasmids pET-*sprha2* and pET-*pbgl* were respectively transformed into *E. coli* BL21 (DE3) to produce proteins.

To construct co-expression plasmids, the DNA fragments of *sprha2* and *pbgl* were inserted into plasmids pET-*pbgl* and pET-*sprha2* respectively by In-fusion clone to produce plasmids pET-*pbgl*-*sprha2* and pET-*sprha2*-*pbgl*. The recombinant plasmids were verified by DNA sequencing. The plasmids pET-*sprha2-pbgl* and pET-*pbgl-sprha2* were transformed into *E. coli* BL21 (DE3) for whole-cell catalysis. The primers were as follows:

pet30-1: 5′-CCACTGAGATCCGGCTGCTAACAAAGCCCGAAAGG-3′;

pet30-2: 5′-TGGTGGTGGTGCTCGAGTGCGGCCGCAAGCTT-3′;

Inf-1: 5′-CTCGAGCACCACCACCAAAGGAGATATACATATGCACCATCA-3′;

sprha2-3: 5′-CAGCCGGATCTCAGTGGTCAGCGGGTCGTGCCCAGCGTGAC-3′; and pbgl-3: 5′-CAGCCGGATCTCAGTGGTCAGCTTCTACGGTATTTCTTGGTTC-3′.

### Production of recombinant proteins

For recombinant protein expression, a single colony was picked and inoculated into a flask containing 10 mL of Luria-Bertani (LB) medium for seed preparations, which included kanamycin (50 mg/L) and cultured overnight at 37 °C with constant shaking at 220 rpm. Then, 4 mL of seed culture was inoculated into 200 mL of LB medium containing kanamycin (50 mg/L) in a 500 mL flask, which was incubated at 37 °C with shaking at 220 rpm. When OD_600nm_ reached 0.4–0.6, isopropyl-β-D-thiogalactoside (IPTG) was added to a final concentration of 0.5 mM to induce recombinant protein expression. To reduce inclusion body formation and improve soluble protein expression, the cells were incubated at 20 °C with shaking at 180 rpm. After 20 h, cells were harvested by centrifugation at 6000×g for 10 min. The cell pellet was resuspended in 10 mL of lysis buffer (50 mM NaH_2_PO_4_, 300 mM NaCl, 10 mM imidazole, pH 8.0) and the cells were lysed by sonication. The lysate was centrifuged at 12,000×g for 30 min. The supernatant was then added to a 1 mL Ni-NTA agarose column (Qiagen, Hilden, Germany). The column was put on ice and gently shaken for 1 h. Then, the column was washed with 6 mL of wash buffer (50 mM NaH_2_PO_4_, 300 mM NaCl, 20 mM imidazole, pH 8.0), and the recombinant protein was eluted with 1 mL of elution buffer (50 mM NaH_2_PO_4_, 300 mM NaCl, 250 mM imidazole, pH 8.0). Purified recombinant protein was desalted using Sephadex™ G-25 (GE Healthcare, Freiburg, Germany), eluted with 1 mL of 100 mM sodium phosphate buffer (pH 7.0). The molecular weight and degree of purification were analyzed by 10% SDS-PAGE.

### Enzyme assays

The activity of SPRHA2 and PBGL were respectively determined using *p*-nitrophenyl-*α*-L-rhamnopyranoside (*p*NPR) and *p*NP-β-D-glucopyranosideat (*p*NPG) at 50 °C, pH 7.5 and 50 °C, pH 8.0 for 20 min and 15 min. The reactions were quenched by 2 M Na_2_CO_3_. One unit of enzyme activity was defined as the amount of enzyme that released 1 µmol of *p*NP per minute.

Substrates epimedin A, B and C, icariin, icariside I and baohuoside I were hydrolyzed by SPRHA2 and PBGL. The reactions contained 0.1 g/L substrate and dilute enzyme and were incubated in 100 mM McIlvaine buffer (pH 7.0) at 37 °C. The reactions were terminated by boiling for 5 min, and the products were analyzed by high-performance liquid chromatography (HPLC).

### Icaritin production from icariin by SPRHA2 and PBGL co-hydrolysis

The enzymatic catalysis mixture (200 µL) contained 100 mM McIlvaine buffer (pH 7.0), 0.2 g/L icariin, 10 mg/L SPRHA2 and 10 mg/L PBGL. It was incubated at 45–70 °C for 10 min to determine the optimum reaction temperature. At the optimal temperature, the influence of pH was investigated in 100 mM McIlvaine buffer (pH 6.5-8.0), 200 mM borate saline buffer (pH 8.0–9.0) and 50 mM glycine-NaOH buffer (pH 9.0–10.0). The reactions were terminated by boiling for 5 min, and the products were analyzed by HPLC.

To decrease the amount of residual reaction intermediates and increase production efficiency, the optimum weight ratio of SPRHA2 and PBGL was investigated. Icariin (0.2 g/L) was reacted by a mixture of two pure enzymes at different weight ratios (total concentration 10 mg/L) at 55 °C, pH 8.5 for 10 min. The reactions were terminated by boiling for 5 min, and the products were analyzed by HPLC.

High concentrations of substrate hydrolysis by SPRHA2 and PBGL was studied under optimal reaction conditions. Because the solubility of icariin in water was low, reactions were shook to enhance hydrolysis. Icariin (25–100 g/L) was catalyzed by 40 mg/L SPRHA2 and 60 mg/L PBGL, reactions (500 µL system) were incubated at 55 °C, pH 8.5 with shaking at 220 rpm for 5 h. The reactions were terminated by boiling for 5 min. The outcomes of reactions were analyzed by HPLC. All assays were performed in triplicate.

### Whole-cell catalysis for icaritin production

The *E. coli* strains containing plasmids pET-*sprha2* and pET-*pbgl* were respectively induced as above, and harvested by centrifugation at 6000×g for 10 min. Then, harvested cells were washed with 0.9% NaCl solution and resuspended in 100 mM sodium phosphate buffer (pH 7.0). The concentration of wet cells was adjusted in an appropriate range for whole-cell catalysis.

To determine the optimal temperature for reaction, the whole-cell reaction system (200 µL) included 1 g/L icariin, 2.5 g/L wet weight SPRHA2 cells, 2.5 g/L wet weight PBGL cells, and 100 mM McIlvaine buffer (pH 7.0). The mixture was incubated at 45–65 °C with shaking at 220 rpm for 10 min. At the optimal temperature, the influence of pH was investigated in 100 mM McIlvaine buffer (pH 6.5-8.0), 200 mM borate saline buffer (pH 8.0–9.0) and 50 mM glycine-NaOH buffer (pH 9.0–10.0). At the optimal temperature and pH, the weight ratio of wet cells of the two strains was investigated: the whole-cell reaction system (200 µL) contained 1 g/L icariin and different weight ratios of wet cells (total wet cell weight: 5 g/L); it was incubated at 55 °C, pH 9.0 with shaking at 220 rpm for 10 min.

High concentration of substrate catalysis was investigated under the optimal reaction conditions. The reaction system (500 µL) included 40 g/L wet weight cells and 50–200 g/L icariin; it was incubated at 55 °C and pH 9.0 with shaking at 220 rpm for 5 h. All reactions were quenched by boiling for 5 min. All assays were performed in triplicate. The results of reactions were analyzed by HPLC.

### Analysis and identification of icaritin

Samples were analyzed and quantified using HPLC (Agilent 1260, USA), equipped with a reversed-phase C18 column (250 × 4.6 mm, 5 μm, Alltech). The samples were diluted by 50% (v:v) acetonitrile, then injected and eluted with a linear gradient of solvent A (H_2_O) and solvent B (acetonitrile) as follows: 32% B (0–5 min), 32–80% B (5–12 min), 80% B (12–17 min), 80%-32% B (17–20 min), and 32% B (20–23 min) at a flow rate of 1 mL/min, and the UV absorption was measured at 270 nm. The column temperature was 35 °C.

After whole-cell catalyzed conversion of 200 g/L icariin into icaritin, the reaction mixture was washed three times with water to remove glucose and rhamnose produced by the reaction. The purified icaritin was freeze-dried and subjected to NMR analysis by Avance III HD600 NMR spectrometer (Bruker, Swiss). DMSO-*d*_*6*_ was used to dissolve the icaritin, which was then transferred to a 5 mm NMR tube for ^13^ C NMR analysis.

## Electronic supplementary material

Below is the link to the electronic supplementary material.

**Additional file 1:Figure S1.** HPLC analysis of icariside I and baohuoside I hydrolysis by SPRHA2 and PBGL; **Figure S2.** HPLC analysis of epimedin A, B, and C hydrolysis by SPRHA2 and PBGL; **Figure S3.** HPLC analysis of the time course of epimedin C hydrolysis by SPRHA2; **Figure S4.** Time course of hydrolysis of whole-cell hydrolysis of icariin; **Figure S5.** SDS-PAGE analysis of pET-*pbgl*-*sprha2* and pET-*sprha2*-*pbgl* in *E. coli*. **Table S1.** NMR data for standard icaritin and icaritin produced

## Data Availability

All materials described within this manuscript, and engineered strains are available on request.
