# Supplementary material for: Highly efficient bioconversion of icariin to icaritin by whole-cell catalysis
Source: Microb Cell Fact. 2023 Apr 4;22:64. doi: 10.1186/s12934-023-02068-4 (PMC10071772; doi:10.1186/s12934-023-02068-4)
Supplement: Supplementary file 1 — Additional file 1:Figure S1. HPLC analysis of icariside I and baohuoside I hydrolysis by SPRHA2 and PBGL; Figure S2. HPLC analysis of epimedin A, B, and C hydrolysis by SPRHA2 and PBGL; Figure S3. HPLC analysis of the time course of epimedin C hydrolysis by SPRHA2; Figure S4. Time course of hydrolysis of whole-cell hydrolysis of icariin; Figure S5. SDS-PAGE analysis of pET-pbgl-sprha2 and pET-sprha2-pbgl in E. coli. Table S1. NMR data for standard icaritin and icaritin produced [file 12934_2023_2068_MOESM1_ESM.docx]

**Supporting information**

**Highly efficient bioconversion of icariin to icaritin by whole-cell catalysis**

Yu Lin ^a,1^, Wen-wen Chen ^a,1^, Bo Ding ^a^, Man Guo ^a^, Meng Liang ^a^, Hao Pang ^b^, Yu-tuo Wei ^a^, Ri-bo Huang ^a,b^, Li-qin Du ^a,^*

^a^ State Key Laboratory for Conservation and Utilization of Subtropical Agro-bioresources, Guangxi Research Center for Microbial and Enzymatic Technology, College of Life Science and Technology, Guangxi University, Daxue Road No. 100, Nanning, Guangxi 530005, China

^b^ Guangxi Key Laboratory of Bio-refinery, National Engineering Research Center for Non-Food Biorefinery, State Key Laboratory of Non-Food Biomass and Enzyme Technology, Guangxi Academy of Sciences, Daling Road No. 98, Nanning, Guangxi 530007, China

^1^ These authors contributed equally to this work.

* Corresponding author

*E-mail address*: duliqin@gxu.edu.cn (**Li-qin Du**)


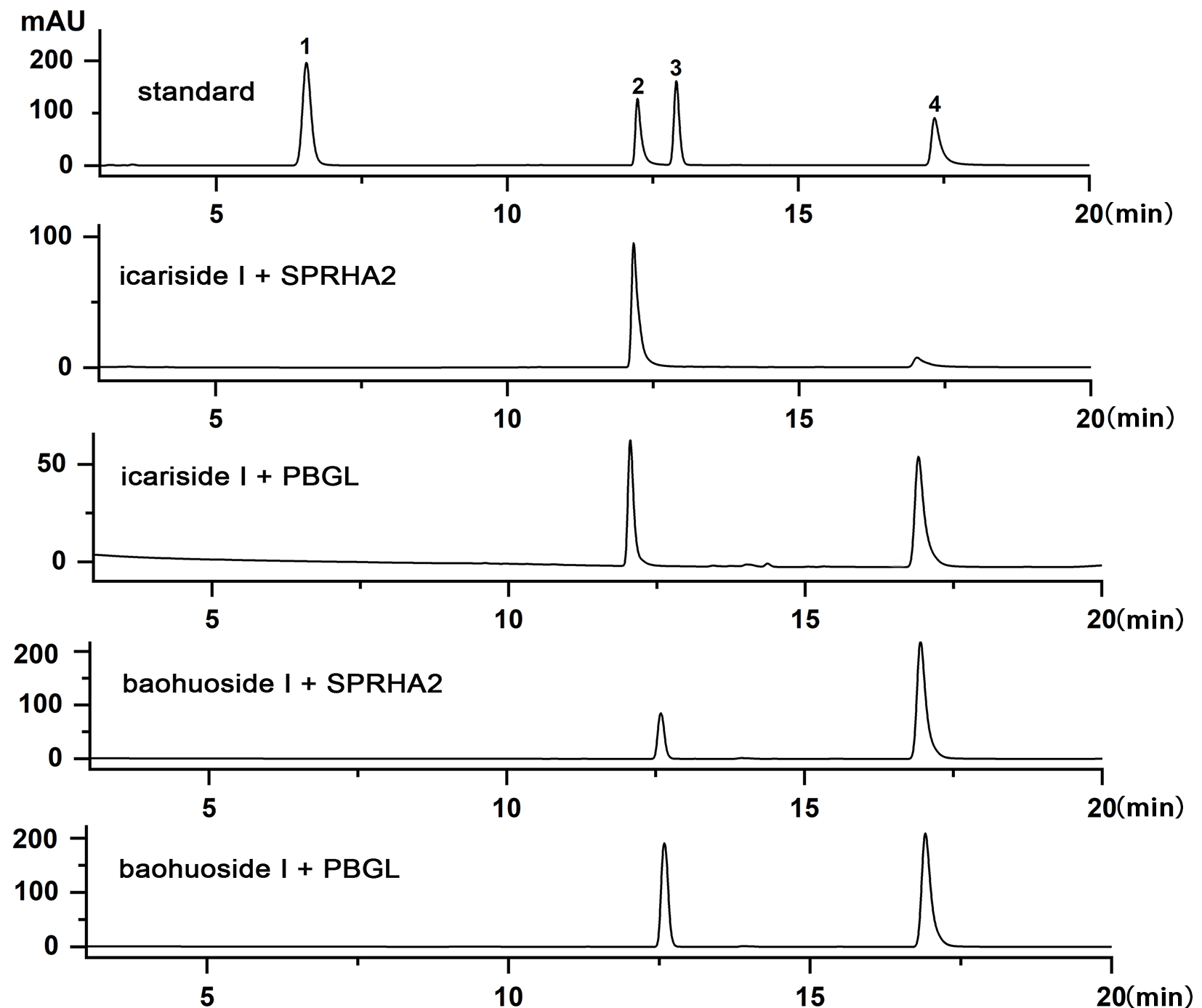


**Figure S1.** HPLC analysis of icariside I and baohuoside I hydrolysis by SPRHA2 and PBGL. Peak 1: icariin; peak 2: icariside I; peak 3: baohuoside I; peak 4: icaritin.


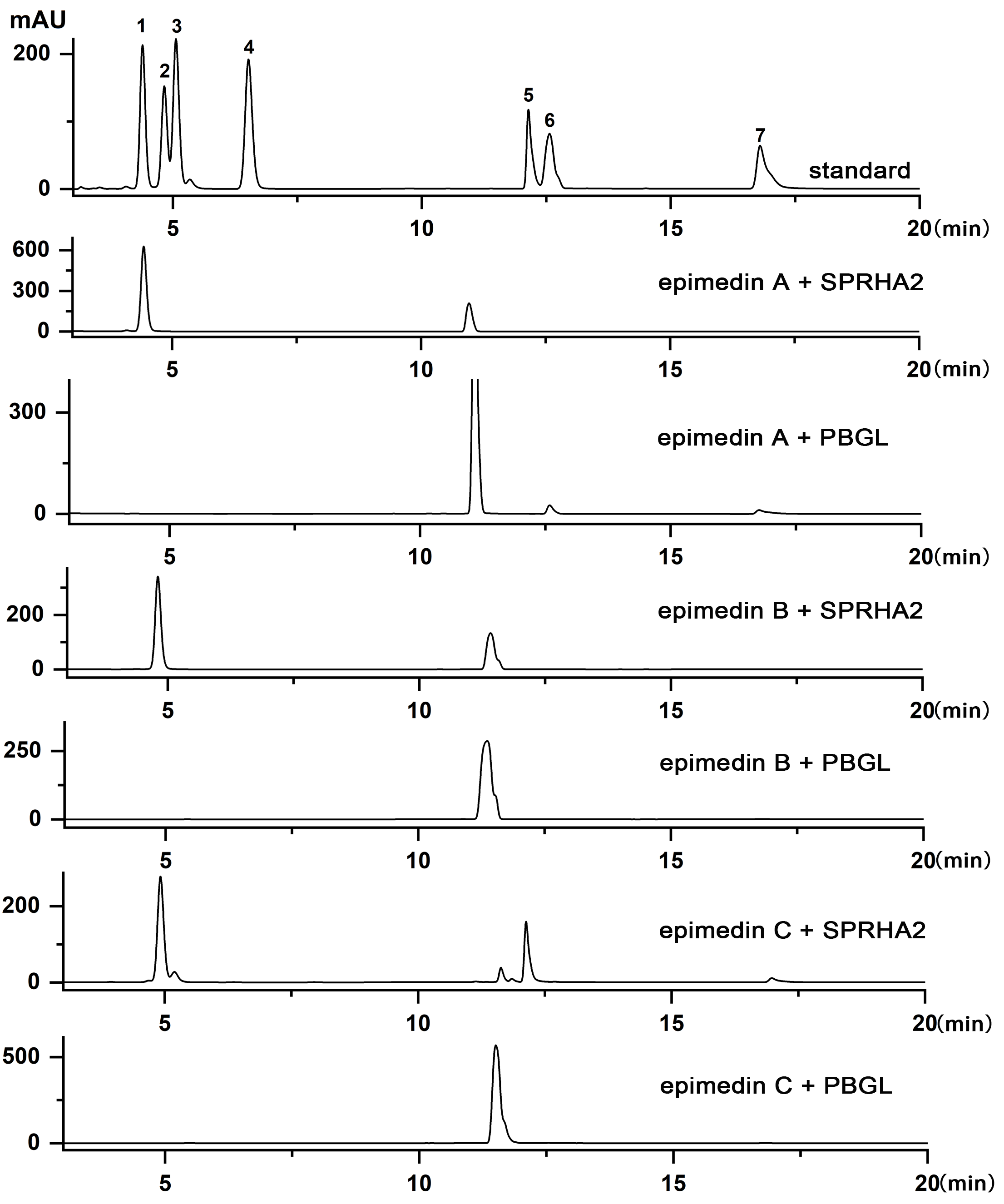


**Figure S2.** HPLC analysis of epimedin A, B, and C hydrolysis by SPRHA2 and PBGL. Peak 1: epimedin A; peak 2: epimedin B; peak 3: epimedin C; peak 4: icariin; peak 5: icariside I; peak 6: baohuoside I; peak 7: icaritin.


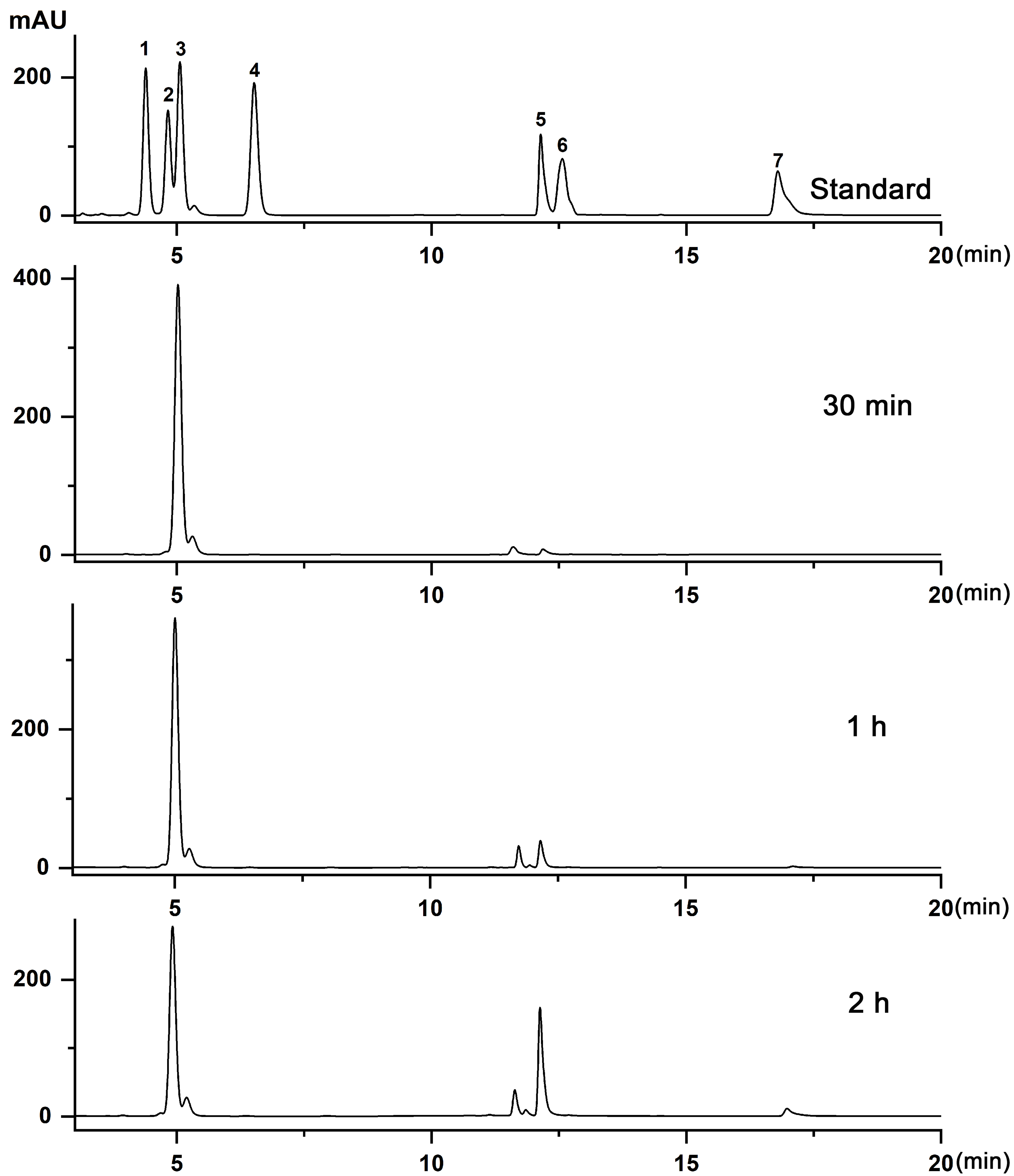


**Figure S3.** HPLC analysis of the time course of epimedin C hydrolysis by SPRHA2. Peak 1: epimedin A; peak 2: epimedin B; peak 3: epimedin C; peak 4: icariin; peak 5: icariside I; peak 6: baohuoside I; peak 7: icaritin.





**Figure S4.** Time course of hydrolysis of whole-cell hydrolysis of icariin.


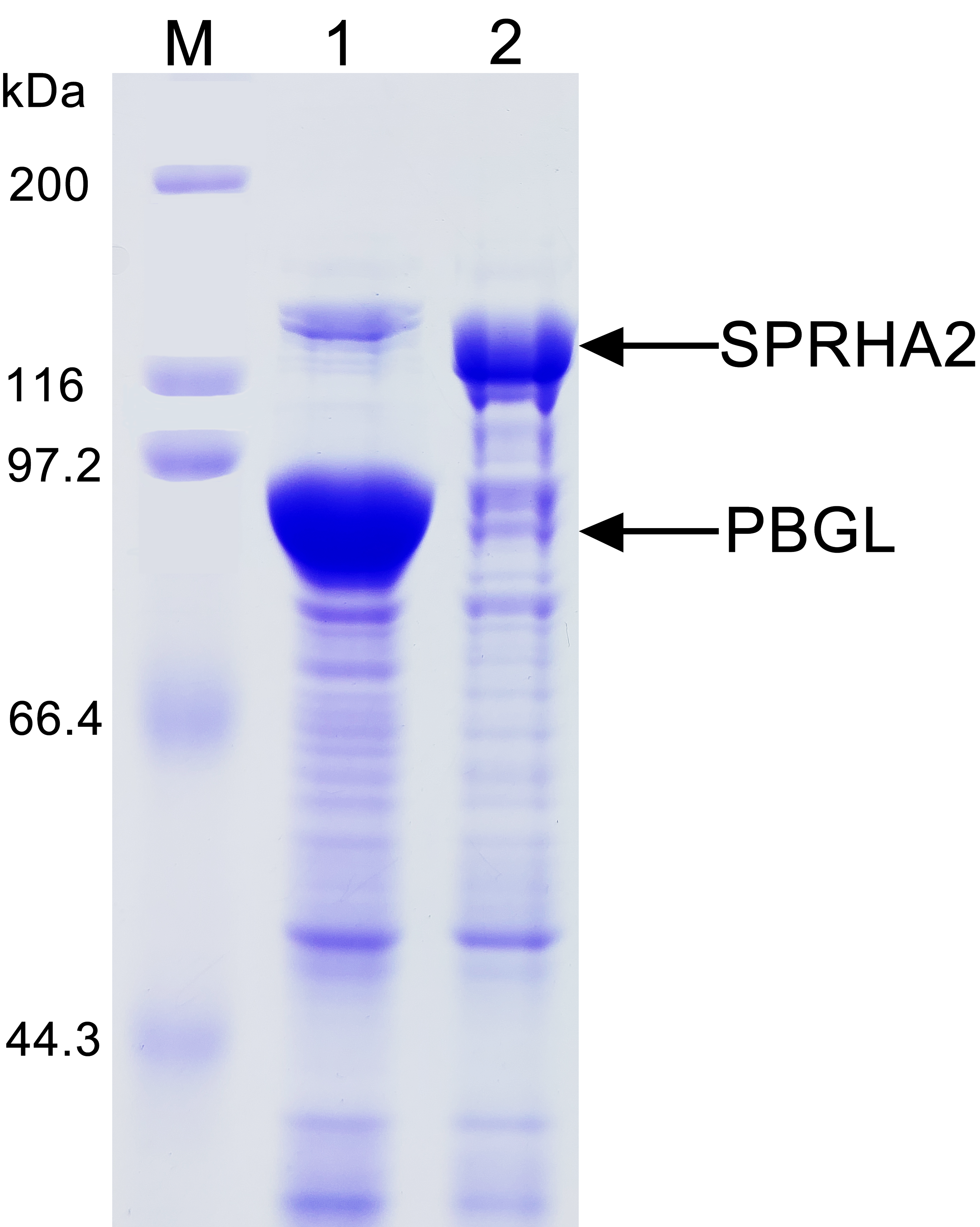
**Figure S5.** SDS-PAGE analysis of pET-*pbgl*-*sprha2* and pET-*sprha2*-*pbgl* in *E. coli*. Lane M: Protein marker; Lane 1: Supernatant of lysate of *E. coli* carrying plasmid pET-*pbgl*-*sprha2*; Lane 2: Supernatant of lysate of *E. coli* carrying plasmid pET-*sprha2*-*pbgl*.

**Table S1**

NMR data for standard icaritin and icaritin produced in the whole-cell reaction system.

| Carbon  no. | DEPT | Icaritin^a^  δc | Product^b^  δc |
| --- | --- | --- | --- |
| 1 | C=O | 176.67 | 176.52 |
| 2 | C | 161.69 | 163.02 |
| 3 | C | 160.93 | 160.83 |
| 4 | C | 158.75 | 158.76 |
| 5 | C | 153.97 | 154 |
| 6 | C | 146.63 | 146.26 |
| 7 | C | 136.37 | 136.37 |
| 8 | C | 131.50 | 131.24 |
| 9 | CH×2 | 129.63 | 129.54 |
| 10 | C | 124.01 | 124.18 |
| 11 | CH | 122.93 | 123.20 |
| 12 | CH×2 | 114.54 | 114.51 |
| 13 | C | 106.1 | 106.09 |
| 14 | C | 103.52 | 103.06 |
| 15 | CH | 98.27 | 98.59 |
| 16 | OCH_3_ | 55.84 | 55.83 |
| 17 | CH_3_ | 25.90 | 25.91 |
| 18 | CH_2_ | 21.66 | 21.17 |
| 19 | CH_3_ | 18.29 | 18.31 |

^a^ Standard icaritin.

^b^ The whole-cell hydrolytic product.
